# Psychological distress and sleep problems when people are under interpersonal isolation during an epidemic: A nationwide multicenter cross-sectional study

**DOI:** 10.1192/j.eurpsy.2020.78

**Published:** 2020-08-28

**Authors:** Shu Wang, Yuan Zhang, Wei Ding, Yao Meng, Huiting Hu, Zhenhua Liu, Xianwei Zeng, Minzhong Wang

**Affiliations:** 1 Department of Neurosurgery, SanBo Brain Hospital, Capital Medical University, Beijing, China; 2 Dalian Medical University, Dalian, China; 3 Department of Pediatric Hematology-Oncology, Dalian Municipal Women and Children’s Medical Center, Dalian, China; 4 Department of Neurology, Liaocheng People’s Hospital, Liaocheng, China; 5 Department of Neurology, The First Affiliated Hospital of Shandong First Medical University, Jinan, China; 6 Department of Neurology, Heze Mudan People’s Hospital, Heze, China; 7 Sleep Medicine Center, Shandong Provincial Hospital Affiliated to Shandong First Medical University, Jinan, China; 8 Department of Neurosurgery, Qilu Hospital of Shandong University, Jinan, China; 9 Department of Neurology, Shandong Provincial Hospital Affiliated to Shandong First Medical University, Jinan, China

**Keywords:** China, COVID-19, cross-sectional study, psychological distress, sleep problems

## Abstract

**Background.:**

During the outbreak of coronavirus disease 2019 (COVID-19), people are under the dual pressure of interpersonal isolation and concerns about infection. An evaluation of people’s psychological status and risk factors is needed to conduct target interventions.

**Methods.:**

This was a nationwide, multicenter, cross-sectional study using quota and snowball sampling methods during the COVID-19 epidemic in China. Participants’ characteristics and experiences were obtained by an online questionnaire and telephone review. Psychological distress and sleep problems were measured by the Generalized Anxiety Disorder-7, the Patient Health Questionnaire-9, and the Insomnia Severity Index.

**Results.:**

A total of 23,500 participants were recruited, and 19,372 valid questionnaires were received from 11 centers. Overall, 11.0–13.3% of the participants had anxiety, depression, or insomnia symptoms, and 1.9–2.7% had severe symptoms. The prevalence of psychological and sleep problems has increased. Working as frontline medical staff (Odds Ratio *OR* = 3.406), living in Hubei Province (*OR* = 2.237), close contacts with COVID-19 (*OR* = 1.808), and age 35–49 years (*OR* = 1.310) were risk factors for anxiety symptoms; no outside activity for 2 weeks (*OR* = 2.167) and age 35–49 years (*OR* = 1.198) were risk factors for depression symptoms; and living in Hubei Province (*OR* = 2.376), no outside activity for 2 weeks (*OR* = 1.927), and age 35–49 years (*OR* = 1.262) were risk factors for insomnia symptoms. Only 1.9% of participants received counseling during the epidemic.

**Conclusions.:**

Psychological and sleep problems increased during interpersonal isolation due to COVID-19. Current psychological interventions are far from sufficient.

## Introduction

Psychological distress and sleep problems are important public health issues when epidemics and disasters occur, and these effects can be long lasting. Previous research has shown that a large proportion of survivors and close contacts of those with Ebola virus disease (EVD) had depression and anxiety symptoms more than two decades after the outbreaks [1]. Similarly, severe acute respiratory syndrome (SARS) and Middle East respiratory syndrome (MERS) had serious psychological influences on a wide range of people [2, 3]. However, most previous studies focused only on infected people or contacts after the epidemic ended. There are few reports on the psychological status of a large sample of people who were isolated during an epidemic outbreak.

In early 2020, an outbreak of coronavirus disease 2019 (COVID-19) began in China, which was caused by a new human-infecting coronavirus [4]. Because the disease was highly contagious and fatal to some patients [5], the Chinese government implemented a nationwide restriction on outside activities following the Spring Festival [6]. People experienced the unique dual pressure of interpersonal isolation and concerns about infection. Although several studies have analyzed psychological health status in China during COVID-19, most of these studies have focused on online social network users and have applied only snowball sampling or convenience sampling, which may decrease their overall representativeness [7–9]. We conducted this large-scale, nationwide, multicenter, cross-sectional study using a population-based representative sampling procedure to explore the psychological impact of interpersonal isolation and the stress of infection among a wide range of people. This study can provide a reference for further psychological intervention during the COVID-19 outbreak and psychological condition models for people experiencing isolation and epidemic stress.

## Methods

### Study population and sampling process

This nationwide, multicenter, cross-sectional study was approved by the SanBo Brain Hospital, Capital Medical University ethics committee. Because face-to-face contact was limited, all participants or their guardians provided informed consent by clicking an “agree to the consent” button online or providing oral consent in a telephone review.

The aim of the current study was to evaluate the psychological status of the general population. The inclusion criteria required all participants to be aged 11 years or older because previous studies and Chinese consensus have only demonstrated the validity of the scales for individuals aged 11 years and older [10]. Participants who refused consent were excluded. The preinvestigation was launched from February 3, 2020 to February 7, 2020, to examine the reliability of the scales, to determine the region-stratified standards, and to foster multicenter collaboration using convenience sampling from several communities. All participants were asked to complete several scales as well as the item, “Which data are most concerning to you during the COVID-19 outbreak?” Response options were as follows: “Number of confirmed patients,” “Number of suspected infections,” “Number of cures,” and “Number of deaths.” A total of 500 participants were recruited and 437 surveys (87.4%) were completed. The number of confirmed patients was the issue of greatest concern (92.0%). Based on the preinvestigation results, areas were stratified by the number of confirmed patients, and psychological distress and sleep problems were measured by the Generalized Anxiety Disorder-7 scale (GAD-7, Cronbach’s *α* = 0.90) [11], the Patient Health Questionnaire-9 (PHQ-9, Cronbach’s *α* = 0.89) [12], and the Insomnia Severity Index (ISI, Cronbach’s *α* = 0.83) [13].

**Figure 1.**
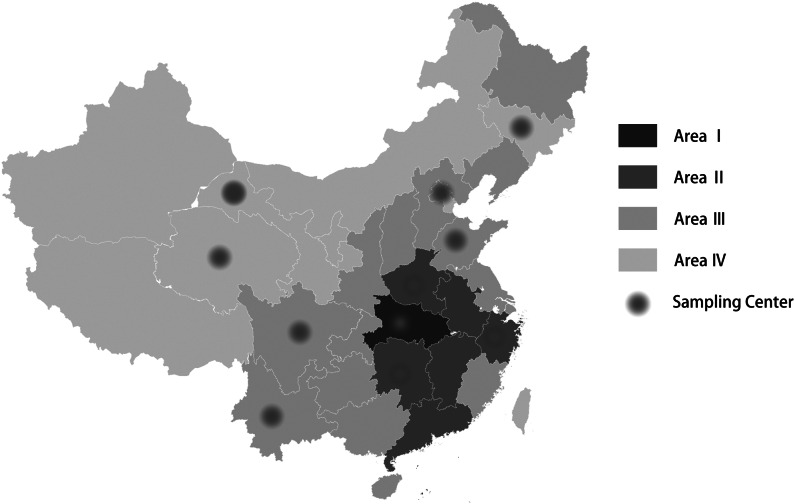
Map of the area division and sampling centers.

**Figure 2.**
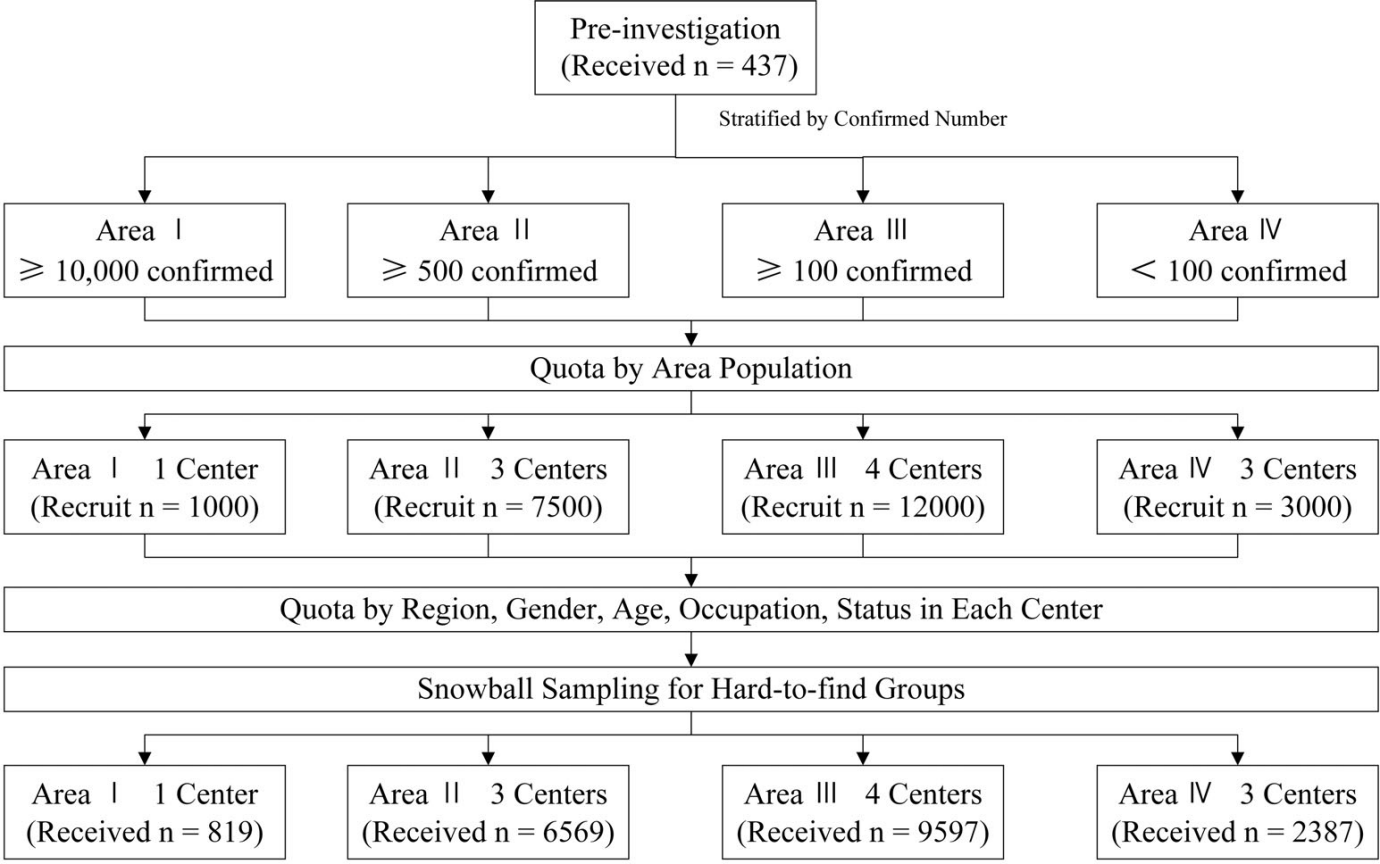
Flow diagram showing the sampling process.

The investigation period was from February 10, 2020 (i.e., 2 weeks after the Spring Festival) to February 17, 2020. The Chinese tradition is to return to one’s registered residence during the Spring Festival; thus, the population is representative of different regions. The government encouraged everyone to stop going outside after returning home from the festival. Because outside activities were restricted, nonprobability quota sampling and snowball sampling methods were used to select representative samples. The Power Analysis and Sample Size (PASS) (NCSS LLC., Kaysville, UT; version 15) software package was used to determine the sample size. According to the preinvestigation, the proportion of anxiety, depression, or insomnia was approximately 0.13, and the dropout rate, confidence level, and permissible error were set at 20, 0.950, and 0.01%, respectively. After calculation, the desired sample size was 17,578, and the desired dropout-inflated enrollment sample size was 21,973. A total of 23,500 participants were planned to be recruited in the formal investigation. According to the data of greatest concern, we stratified the country into four areas by the number of confirmed patients on February 9, 2020 (Area I: ≥ 10,000 confirmed; Area II: ≥ 500 confirmed; Area III: ≥ 100 confirmed; Area IV: < 100 confirmed) based on the report of the National Health Commission of China [14]. The area division and sampling centers are shown in Figure 1. Quotas were determined based on the proportion of population in different areas (Area I: 1 center, 1,000 recruited; Area II: 3 centers, 7,500 recruited; Area III: 4 centers, 12,000 recruited; Area IV: 3 centers, 3,000 recruited) and further stratified by the proportion of population in different sampling centers in the area based on data from the Sixth China National Census [15]. This step was based on the proportion of the population in different areas to realize population-based sampling. For example, assuming that the proportion of these areas was I:II:III:IV = 1:2:2:1; the required number of samples was 600; therefore, in Areas I, II, III, and IV, 100, 200, 200, and 100 people were recruited, respectively. Assuming that Area I had two centers, the proportion of these centers was I:II = 1:1, so each center recruited 50 people. Each center conducted a quota survey based on the region, gender, age, occupation, and status of the people in the area (each center was responsible for one province) based on the proportion of different characteristics, and invitations were sent to these groups of people. Only invited groups of people could participate. This selection procedure was not based on households to realize quota sampling for the representation of entire populations. Because it was difficult to obtain participants with certain occupations and identities (e.g., farmers, confirmed patients), snowball sampling was used as a supplement in each center. When the sampling center could not recruit enough people with the required characteristics after 5 days, 2 days were used to ask participants to introduce others with the required characteristics to recruit specific groups. The sampling centers were required to use quota sampling whenever possible; snowball sampling was expected to not exceed 5% of the samples. When the center recruited enough participants (as the quotas required), the recruitment procedure was finished. Figure 2 shows the flow diagram of the sampling process.

### Measures

After the participants were recruited, another review team conducted the investigation through an online questionnaire and telephone review (for people who could not finish the online questionnaire, the contents of the telephone review were the same as the online questionnaire and were read by the investigators). Each participant was required to answer only once by one method (online or telephone). Network IP address restrictions were set to prevent multiple answers from the same person. A token payment was used as a recruitment incentive for all recruited participants after responding, whether valid or not. The amount of payment and proposed method and timing of disbursement were approved by the ethics committee. The questionnaire consisted of three parts, and participants were asked to respond based on the past 2 weeks. The first part was the characteristics of the participants, including area, age, gender, occupation, education, and marriage. The second part was experience of the epidemic, including “status of COVID-19 (S1: cured patients; S2: confirmed patients; S3: suspected infection; S4: close contacts except frontline medical staff; S5: frontline medical staff; S6: others, i.e., noncontacts),” “outside activity in the past 2 weeks (never in the past 2 weeks; once in 8 days or more; once in 1–7 days),” “counseling during the COVID-19 outbreak (yes; no),” and “similar memory of epidemic experiences (SARS, severe acute respiratory syndrome, outbreak in 2003, China; H1N1, H1N1 flu, outbreak in 2009, China; both; none).” The third part was the GAD-7, the PHQ-9, and the ISI scales. The GAD-7 (scored 0–21) is a 7-item self-report scale for rapid assessment of anxiety symptoms; higher scores indicate a higher likelihood of having severe anxiety symptoms. A score ≥10 indicates anxiety symptoms, and ≥15 indicates severe symptoms. The PHQ-9 (scored 0–27) is a 9-item self-report scale used to assess depression symptoms. Participants with higher scores are more likely to have severe depression symptoms. Depression symptoms are indicated by scores ≥10 for adults and ≥11 for those aged under 18 [16]. Scores ≥15 indicate severe depression symptoms. The ISI (scored 0–28) has 7 self-reported questions to assess sleep condition. Higher scores indicate a higher likelihood of having severe insomnia symptoms. A total ISI score ≥15 indicates insomnia symptoms, and ≥22 indicates severe insomnia symptoms. All scales and classification standards adopted were Chinese revised versions that were reviewed by neuropsychologists. In addition, a 1-item general trust question was used at the end, which was recommended by Chinese versions of the scales for network or telephone reviews. Participants were asked, “Did you answer truthfully?” Questionnaires with “no” responses on the trust question, incomplete answers, and short response times (i.e., less than 1 min) were considered invalid.

### Statistical analysis

Continuous variables were presented as the mean ± standard deviation or ranges and interquartile ranges (IQRs). Categorical variables were reported as a number and percentage. For risk factor analyses of psychological distress and sleep problems, continuous variables were stratified, and the cut-off values were determined by demographics. The division of ages followed a previous large-scale Chinese epidemiological study of mental health by Huang et al. during a normal period, which was set as a contrast to explore changes during the epidemic [17]. Univariate analyses and comparisons of categorical variables were performed using univariate logistic regression. Variables showing *p* < 0.05 in the univariate analysis were entered into the multiple logistic regression model in a backward fashion to adjust for the confounding effects of other variables. In addition, Pearson’s correlation analysis was performed to explore the correlation of scores of different scales. Statistical significance was defined as *p* < 0.05. Statistical analyses were performed using SPSS (IBM, Armonk, NY; version 26).

## Results

### Characteristics of the participants

In accordance with the sampling strategy, a total of 23,500 participants were recruited, and a total of 19,372 valid questionnaires (18,791 from quota sampling, 97.0%, and 581 from snowball sampling, 3.0%) were received from 11 centers (Area I: 819; Area II: 6,569; Area III: 9,597; Area IV: 2,387). The overall valid response rate was 82.4%. Of the excluded samples, 749 did not provide consent, 1,610 did not complete the full questionnaire, 803 did not pass the trust question, and 966 answered in short response times. The proportion of age, gender, occupation, regional population, and status of participants did not differ significantly from the proposal and the Sixth China National Census [15] (*p* > 0.05), suggesting that the drop-out samples did not significantly influence the proportion of the samples’ characteristics. The age of participants at the time of the survey ranged from 11 to 87 years (IQR 22–61); 48.0% (9,307) of participants were male, and 52.0% (10,065) were female. The basic characteristics are shown in Table 1. Supplementary Table S1 shows the detailed information.

**Table 1.** Participants’ characteristics (*n* = 19,372).

Subject	*N*	%
Area		
I	819	4.23
II	6,569	33.91
III	9,597	49.54
IV	2,387	12.32
Age, years		
10–17	3,306	17.07
18–34	4,582	23.65
35–49	4,307	22.23
50–64	3,617	18.67
≥65	3,560	18.38
Gender		
Male	9,307	48.04
Female	10,065	51.96

Note: Refer to Supplementary Table S1 for detailed characteristics.

Participants’ experience with the COVID-19 was also assessed. Because the number of cures was limited at the time of sampling, no cured patients were included. A total of 181 (0.9%) confirmed patients, 218 (1.1%) suspected infections, 301 (1.6%) close contacts, and 179 (0.9%) frontline medical staff completed the questionnaire. According to the survey, 9,745 (50.3%) participants had no outdoor activity in the past 2 weeks. Only 376 (1.9%) participants received counseling or psychotherapy. Supplementary Table S2 shows the participants’ experience of the epidemic in detail.

### Psychological distress

Overall, 12.2% (*n* = 2,372, 95% Confidence Interval CI: 11.8–12.7) of the participants had anxiety symptoms and 2.3% (*n* = 445, 95% CI: 2.1–2.5) had severe anxiety symptoms (scores ≥15) based on the GAD-7. In the univariate analysis (Supplementary Tables S1 and S2), the following factors were associated with anxiety symptoms: area, age, occupation, COVID-19 status, and similar memories. Multivariate analysis of statistically significant factors in the univariate analysis showed that frontline medical staff (*OR* = 3.406, 95% CI: 2.480–4.679), Area I (*OR* = 2.237, 95% CI: 2.012–2.510), close contacts except frontline medical staff (*OR* = 1.808, 95% CI: 1.115–2.539), and age 35–49 years (*OR* = 1.310, 95% CI: 1.187–1.446) were independent risk factors for anxiety symptoms (Table 2).

**Table 2.** Overall risk factors of psychological distress and sleep problems by multivariate analyses.

Scale	Variables	*OR*	95% CI	*p*
GAD-7	Area I	2.237	2.012–2.510	0.037
	Age 35–49 years	1.310	1.187–1.446	0.018
	Close contacts^a^	1.808	1.115–2.539	0.029
	Frontline medical staff	3.406	2.480–4.679	0.003
PHQ-9	Age 35–49 years	1.198	1.079–1.329	0.024
	No outside activity for 2 weeks	2.167	2.067–2.278	0.022
ISI	Area I	2.376	2.142–2.658	0.015
	Age 35–49 years	1.262	1.147–1.389	0.019
	No outside activity for 2 weeks	1.927	1.037–2.224	0.025

Adjusted for all other variables. The contrast was set as indicator determined by the group with lowest prevalence of anxiety, depression, or insomnia to explore the risk factors.

Abbreviations: GAD-7, the Generalized Anxiety Disorder-7 scale; ISI, the Insomnia Severity Index; PHQ-9, the Patient Health Questionnaire-9; SARS, severe acute respiratory syndrome, outbreak in 2003, China.

a Except for frontline medical staff.

According to the PHQ-9, 11.0% (*n* = 2,138, 95% CI: 10.6–11.5) of the participants were likely to have depression symptoms and 1.9% (*n* = 368, 95% CI: 1.7–2.1) had severe depression symptoms (scores ≥15). Univariate analysis revealed that area, age, gender, marriage status, and outside activity status were associated with depression symptoms. Multivariate analysis revealed that no outside activity for 2 weeks (*OR* = 2.167, 95% CI: 2.067–2.278) and age 35–49 years (*OR* = 1.198, 95% CI: 1.079–1.329) were risk factors for depression symptoms.

### Sleep problems

The results showed that 13.3% (*n* = 2,577, 95% CI: 12.8–13.8) of participants had ISI scores ≥15, which indicated that they had insomnia symptoms, and 2.7% (*n* = 523, 95% CI: 2.5–2.9) of participants had ISI scores ≥22, indicating severe insomnia symptoms. The univariate analysis showed that area, age, occupation, and outside activity status were associated with insomnia symptoms. The multivariate analysis showed that Area I (*OR* = 2.376, 95% CI: 2.142–2.658), no outside activity for 2 weeks (*OR* = 1.927, 95% CI: 1.037–2.224), and age 35–49 years (*OR* = 1.262, 95% CI: 1.147–1.389) were risk factors for insomnia symptoms.

To explore psychological distress and sleep problems and their risk factors in different regions and statuses during the epidemic, samples were further stratified by region and status. Univariate and multivariate analyses were performed for every subgroup (Table 3). The correlation of these three scales was analyzed, and the scores of anxiety and insomnia symptoms were positively correlated in these samples (*r* = 0.683, *p* = 0.012). The correlation between scores for depression and insomnia symptoms and for anxiety and depression symptoms did not reach statistical significance (*r* = 0.519, *p* = 0.064; *r* = 0.492, *p* = 0.152).

**Table 3.** Risk factors of psychological distress and sleep problems (stratified by area or status) by multivariate analyses.

Area	Scale	Variables	*OR*	95% CI	*p*
Stratified by area					
Area I	GAD-7	Age 35–49 years	2.042	1.131–3.257	0.029
		Frontline medical staff	4.016	2.137–6.271	0.002
		Close contacts^a^	1.808	1.115–2.539	0.017
Area IV	GAD-7	Age 35–49 years	1.263	1.026–1.329	0.024
		Frontline medical staff	2.782	1.384–3.873	0.003
	PHQ-9	Age 35–49 years	1.253	1.083–1.582	0.015
		No outside activity for 2 weeks	2.173	1.482–2.934	0.009
Stratified by status					
S3	ISI	Area I	2.053	1.259–3.383	0.016
		Similar memory of SARS only	1.933	1.026–2.365	0.023
S6	GAD-7	Area I	2.229	2.068–2.475	0.029
		Age 35–49 years	1.424	1.192–1.986	0.016
	PHQ-9	Age 35–49 years	1.177	1.012–1.229	0.044
		No outside activity for 2 weeks	2.091	1.825–2.428	0.020

Adjusted for all other variables. The contrast was set as indicator determined by the group with lowest prevalence of anxiety, depression, or insomnia to explore the risk factors.

Abbreviations: GAD-7, the Generalized Anxiety Disorder-7 scale; ISI, the Insomnia Severity Index; PHQ-9, the Patient Health Questionnaire-9; S3, Suspected infection; S4, Close contacts (Except frontline medical staff); S6, Others; SARS, severe acute respiratory syndrome, outbreak in 2003, China.

a Except for frontline medical staff.

## Discussion

Anxiety and depression are common psychological problems after disasters and epidemics. Previous studies suggest that 30–37% of survivors and contacts of epidemics (e.g., EVD, SARS, MERS) have anxiety and depression [1,2,18]. Disaster experiences such as earthquakes are also associated with depression and anxiety [19–21]. Poor sleep quality is a common manifestation of anxiety and depression. Insomnia has been identified as a predictor of anxiety and depression [22]. Previous postdisaster studies suggest that sleep disturbances are common in disaster survivors and can be long lasting [23–25]. Furthermore, the economic impacts of an epidemic because of cross-regional trade reduction and factory closures influence individuals [26]. Periods of economic recession are thought to be associated with a higher prevalence of mental health problems [27]. Therefore, nationwide psychological and sleep problems during the outbreak need to be assessed.

The present study found that 11.0–13.3% of the participants had anxiety, depression or insomnia symptoms and that 1.9–2.7% had severe psychological distress or sleep problems. According to Huang et al.’s epidemiological report of the prevalence of mental disorders in China, the 12-month prevalence of anxiety disorders and depression disorders was 5.0% (95% CI: 4.2–5.8) and 3.6% (95% CI: 3.0–4.2), respectively [17]. Another cross-sectional study based on a community-based population in China showed that 8.7% (95% CI: 8.2–9.3) of the participants had insomnia [28]. The prevalence of psychological and sleep problems increased during the outbreak of COVID-19. Although the scales used in the present study were only screening assessments, they indicate a higher prevalence of psychological problems among the participants. Several studies have shown that 16.5–28.8% of people had psychological health problems during the outbreak of the COVID-19 epidemic in China, which is higher than our findings [7–9]. A survey based on Chinese online social network software showed that nearly one-third of people had anxiety symptoms during the COVID-19 outbreak [7]. However, because these studies only investigated network users and used only snowball or convenience sampling, most of the participants may have been concerned about the network and the epidemic, which could explain the higher proportion of psychological problems than found in our study. Additionally, because these studies were restricted to web users, the participants were not representative of all age groups [9]. The present study used a population-based representative sampling procedure and considered people of different ages, genders, occupations, and epidemic statuses to provide a comprehensive reference for overall psychological status and further interventions. In addition, the present study had a high valid response rate (82.4%), which might be because of the recruitment and assessment procedure and the recruitment incentive.

Area I (i.e., Hubei Province) is a high incidence area for anxiety and insomnia symptoms. Hubei Province (including Wuhan) was considered the earliest location of the outbreak of COVID-19 in China [29]. On January 23, 2020, due to epidemic control, the Wuhan government adopted strict measures to prohibit citizens from leaving Wuhan, and all air travel, train travel and public transportation were canceled, which caused people in this region to experience the dual pressures of panic and isolation earlier than other regions [30]. The present research suggests that residents of Hubei Province have significantly higher levels of anxiety and insomnia symptoms than other regions, and efforts should be made to strengthen psychological counseling while controlling the epidemic. The current study did not find an exceedingly higher proportion of psychological and sleep problems in Hubei Province than in other regions, as in previous studies [7,9], which might be due to different sampling methods. Because Wuhan Province was still under strict epidemic control during the research period, the current research covered only a limited number and range of samples. A more thorough study is needed when possible.

According to Huang et al.’s study, individuals aged 35–49 years old have a lower prevalence of mood disorder (3.8%) (e.g., depression, anxiety) than other adult groups (3.9–4.5%) in China [17]. However, the present study found that participants aged 35–49 years had more severe anxiety, depression, and insomnia symptoms during the outbreak of COVID-19. Early reports and studies suggested that middle-aged and elderly people may be more susceptible to COVID-19 [5], but elderly people have less outdoor activity. Individuals aged 35–49 years old have more outside cross-regional activities for work reasons, which may increase their concerns about infection. In China, individuals aged 35–49 years old often hold important positions in companies and families, and the economic impact of the epidemic may increase their concerns [31]. These factors increase the likelihood that people in this age group may have mental disorders that need to be treated. Furthermore, because elderly people had a lower valid response rate, young and middle-aged people mainly contributed to the sample group (ages IQR 22–61 years). The evaluation of psychological status in the elderly group requires further exploration and analyses. It should also be noted that new epidemic information may inform people in the future and change people’s understanding. Updated studies are needed to examine this issue.

Close contacts of people affected by COVID-19 and frontline medical staff were found to be more likely to have anxiety symptoms. Previous reports suggest that close contacts and medical staff had anxiety, depression, and even post-traumatic stress disorder (PTSD) after the epidemic outbreak [1,2,18]. This study suggests that close contacts and frontline medical staff are at higher risk of anxiety during the outbreak. To manage long-term isolation and epidemic control, psychological support for these groups should be provided as soon as possible. Purssell et al. conducted a meta-analysis and suggested that compared with non-isolated patients, isolated patients showed higher levels of depression and higher levels of anxiety [32]. In the present study, after 2 weeks of strict restrictions on outside activity, people who did not have any outside activity for 2 weeks had a higher proportion of depression and insomnia. The lack of interpersonal communication and the panic of the epidemic led to a greater need for psychological intervention in this group. However, the present study did not find that confirmed patients and suspected infections were risk factors for anxiety, depression, or insomnia symptoms, which may be related to the limited sample collection.

In the present study, only 1.9% (376) of the participants received counseling during the COVID-19 outbreak, which is not enough for the potential proportion of psychological problems. Inadequate attention to mental health, a lack of systematic training and the scarcity of professional practitioners remain challenges [33]. Additionally, outdoor restrictions may explain the low percentage of participants who reported receiving counseling. The outbreak limits face-to-face counseling and individualized treatment. Some studies suggest that online or telephone counseling may be helpful for treatment during epidemic outbreaks [34]. A study of telephone consultations in Hong Kong during the SARS outbreak reported that people’s levels of anxiety could be decreased significantly after telephone health education [35]. However, non-face-to-face psychological counseling and guidance may have limited reliability in disease assessment and individualized treatment [36], which requires further research. Cipolletta et al. reviewed the attitudes of psychologists towards different aspects of online counseling and expressed concerns about online diagnosis and therapeutic interventions. They also found a consistent lack of clarity regarding ethical issues with regard to online modalities [37]. Public psychological health education and self-screening are also convenient ways to improve psychological health and identify problems early. These approaches can be conducted through non-face-to-face methods for large groups of people [38]. We suggest that under the current circumstances, psychological intervention using all possible methods are necessary and urgent for mental illness outbreaks during and after the epidemic. More research is needed to confirm the efficacy of different intervention methods. Moreover, additional efforts should be made to conduct targeted psychological interventions for specific populations with a higher risk of psychological problems based on epidemiological investigations.

Future studies should also focus on PTSD. Previous research found that survivors of a disaster or epidemic have a high probability of experiencing PTSD. Wu et al. [2] found that approximately 10% of hospital employees experienced high levels of PTSD symptoms 3 years after the SARS outbreak in China. Compared to other groups, participants who had been quarantined, worked in high-risk locations such as SARS wards, or had friends or close relatives who contracted SARS were 2 to 3 times more likely to have high PTSD levels. A cross-sectional study of the general population in Sierra Leone 1 year after the EVD outbreak in 2015 showed that the prevalence of anxiety-depression symptoms was 48% and the prevalence of any PTSD symptom was 76% [39]. Chronic fatigue after an epidemic can have an indirect long-term effect on PTSD through persistent depression in MERS survivors [40]. People with experience related to COVID-19 may also need psychological support. Future research should follow survivors to understand the impact of PTSD and determine appropriate treatment options.

### Limitations

First, due to restrictions on outside activities, random sampling could not be used in this study. The nonprobability sampling method may not accurately represent the entire population. To improve overall representation, we adopted multicenter large-scale sampling and performed population-based stratification and quota sampling. Because nonrandom resampling was adopted and some participants did not respond, selection bias was inevitable. A random sampling study should be conducted after the outbreak has been controlled. Second, due to the limited number of cures during the survey period, no cured participants were included, and some subsamples were small (e.g., confirmed patients, elderly people). Given the limitations of the scale criteria, only participants aged 11 years or older were included in the present study. Further research should also consider the psychological health of children under 11 years of age. Finally, as a cross-sectional study, changes in the prevalence and severity of psychological symptoms and changes in the epidemic situation and people’s recognition cannot be identified. This issue requires further follow-up studies.

## Conclusions

The present study found that 11.0–13.3% of participants had anxiety, depression, or insomnia symptoms and that 1.9–2.7% had severe psychological distress or sleep problems during the outbreak of COVID-19. Due to interpersonal isolation and the stress of infection, the prevalence of psychological problems increased. Working as frontline medical staff (*OR* = 3.406), living in Hubei Province (*OR* = 2.237), close contacts with COVID-19 (*OR* = 1.808), and age 35–49 years (*OR* = 1.310) were risk factors for anxiety symptoms; no outside activity for 2 weeks (*OR* = 2.167) and age 35–49 years (*OR* = 1.198) were risk factors for depression symptoms; and living in Hubei Province (*OR* = 2.376), no outside activity for 2 weeks (*OR* = 1.927), and age 35–49 years (*OR* = 1.262) were risk factors for insomnia symptoms. Only 1.9% of the participants received counseling during the epidemic. These groups require more attention to their psychological health. The current psychological interventions are far from sufficient.

## Data Availability

All characteristics of the samples and preliminary analysis results have been uploaded in detail as Supplementary Material. Because the investigation procedure collected some personal information (e.g., location, IP address, telephone number, etc.), the informed consent and the ethical agreement did not allow sharing of detailed questionnaire raw data to public or third party for personal privacy protection.

## Supplementary material

For supplementary material accompanying this paper visit http://dx.doi.org/10.1192/j.eurpsy.2020.78. click here to view supplementary material

## Abbreviations

COVID-19: coronavirus disease 2019
EVD: Ebola virus disease
GAD-7: the Generalized Anxiety Disorder-7 scale
H1N1: H1N1 flu
ISI: the Insomnia Severity Index
PHQ-9: the Patient Health; Questionnaire-9
SARS: severe acute respiratory syndrome
MERS: Middle East respiratory syndrome
