# Supplementary material for: Psychological distress and sleep problems when people are under interpersonal isolation during an epidemic: A nationwide multicenter cross-sectional study
Source: Eur Psychiatry. 2020 Aug 28;63(1):e77. doi: 10.1192/j.eurpsy.2020.78 (PMC7503168; doi:10.1192/j.eurpsy.2020.78)
Supplement: Supplementary file 1 [file S0924933820000784sup.zip › S0924933820000784sup002.docx]

**Supplementary Table S2. Participants’ experience and overall risk factors of psychological distress and sleep problems by univariate analyses.**

| **GAD-7** | | | | **PHQ-9** | | | | **ISI** | | | |
| --- | --- | --- | --- | --- | --- | --- | --- | --- | --- | --- | --- |
| **Experience** | **Scores ≥10** | **Scores <10** | ***p*** | **Experience** | **Scores ≥10*** | **Scores <10** | ***p*** | **Experience** | **Scores ≥15** | **Scores <15** | ***P*** |
| Status ^a^ |  |  |  | Status ^a^ |  |  |  | Status ^a^ |  |  |  |
| S2, n = 181 | 24 (13.26) | 157 (86.74) | <0.001^c^ | S2, n = 181 | 22 (12.15) | 159 (87.85) | 0.358 | S2, n = 181 | 25 (13.81) | 156 (86.19) | 0.151 |
| S3, n = 218 | 31 (14.22) | 187 (85.78) |  | S3, n = 218 | 26 (11.93) | 192 (88.07) |  | S3, n = 218 | 32 (14.68) | 186 (85.32) |  |
| S4, n = 301 | 52 (17.28) | 249 (82.72) |  | S4, n = 301 | 36 (11.96) | 265 (88.04) |  | S4, n = 301 | 54 (17.94) | 247 (82.06) |  |
| S5, n = 179 | 57 (31.84) | 122 (68.16) |  | S5, n = 179 | 23 (12.85) | 156 (87.15) |  | S5, n = 179 | 23 (12.85) | 156 (87.15) |  |
| S6, n = 18493 | 2208 (11.94) | 16285 (88.06) |  | S6, n = 18493 | 2031 (10.98) | 16462 (89.02) |  | S6, n = 18493 | 2443 (13.21) | 16050 (86.79) |  |
| Outside activity |  |  |  | Outside activity |  |  |  | Outside activity |  |  |  |
| Never, n = 9745 | 1196 (12.27) | 8549 (87.73) | 0.718 | Never, n = 9745 | 1149 (11.79) | 8596 (88.21) | 0.003^c^ | Never, n = 9745 | 1363 (13.99) | 8382 (86.01) | 0.016^b^ |
| 1-7 d, n = 872 | 103 (11.81) | 769 (88.19) |  | 1-7 d, n = 872 | 95 (10.89) | 777 (89.11) |  | 1-7 d, n = 872 | 105 (12.04) | 767 (87.96) |  |
| 8- d, n = 8755 | 1042 (11.90) | 7713 (88.10) |  | 8- d, n = 8755 | 894 (10.21) | 7861 (89.79) |  | 8- d, n = 8755 | 1109 (12.67) | 7646 (87.33) |  |
| Counselling |  |  |  | Counselling |  |  |  | Counselling |  |  |  |
| Yes, n = 376 | 36 (9.57) | 340 (90.43) | 0.132 | Yes, n = 376 | 36 (9.57) | 340 (90.43) | 0.361 | Yes, n = 376 | 44 (11.70) | 332 (88.30) | 0.356 |
| No, n = 18996 | 2305 (12.13) | 16691 (87.87) |  | No, n = 18996 | 2102 (11.07) | 16894 (88.93) |  | No, n = 18996 | 2533 (13.33) | 16463 (86.67) |  |
| Sim. Memory |  |  |  | Sim. Memory |  |  |  | Sim. Memory |  |  |  |
| SARS, n = 763 | 108 (14.15) | 655 (85.85) | 0.002^c^ | SARS, n = 763 | 84 (11.01) | 679 (88.99) | 0.256 | SARS, n = 763 | 107 (14.02) | 656 (85.98) | 0.546 |
| H1N1, n = 1036 | 124 (11.97) | 912 (88.03) |  | H1N1, n = 1036 | 109 (10.52) | 927 (89.48) |  | H1N1, n = 1036 | 126 (12.16) | 910 (87.04) |  |
| Both, n = 14832 | 1835 (12.37) | 12997 (87.63) |  | Both, n = 14832 | 1652 (11.14) | 13180 (88.86) |  | Both, n = 14832 | 1991 (13.42) | 12841 (86.58) |  |
| None, n = 2741 | 274 (10.00) | 2467 (90.00) |  | None, n = 2741 | 293 (10.69) | 2448 (89.31) |  | None, n = 2741 | 353 (12.88) | 2388 (87.12) |  |

GAD-7: the Generalized Anxiety Disorder-7 scale; PHQ-9: the Patient Health Questionnaire-9; ISI: the Insomnia Severity Index; S2: Confirmed patients; S3: Suspected infection; S4: Close contacts (Except frontline medical staff); S5: Frontline medical staff; S6: Others, i.e. non contacts; SARS: Severe Acute Respiratory Syndrome, outbreak in 2003, China; H1N1: H1N1 Flu, outbreak in 2009, China. * For participants aged under 18, scores ≥11 indicate depression; ^a^ No S1 (Cured patients) participant recruited; ^b^ *p* < 0.05 (Univariate logistic regression); ^c^ *p* < 0.01 (Univariate logistic regression).
