# Supplementary material for: Psychological distress and sleep problems when people are under interpersonal isolation during an epidemic: A nationwide multicenter cross-sectional study
Source: Eur Psychiatry. 2020 Aug 28;63(1):e77. doi: 10.1192/j.eurpsy.2020.78 (PMC7503168; doi:10.1192/j.eurpsy.2020.78)
Supplement: Supplementary file 1 [file S0924933820000784sup.zip › S0924933820000784sup001.docx]

**Supplementary Table S1. Participants’ characteristics and overall risk factors of psychological distress and sleep problems by univariate analyses.**

| **GAD-7** | | | | **PHQ-9** | | | | **ISI** | | | |
| --- | --- | --- | --- | --- | --- | --- | --- | --- | --- | --- | --- |
| **Characteristics** | **Scores ≥10** | **Scores <10** | ***p*** | **Characteristics** | **Scores ≥10*** | **Scores <10** | ***p*** | **Characteristics** | **Scores ≥15** | **Scores <15** | ***P*** |
| Area |  |  |  | Area |  |  |  | Area |  |  |  |
| Ⅰ, n = 819 | 118 (14.41) | 701 (85.59) | 0.003^b^ | Ⅰ, n = 819 | 106 (12.94) | 713 (87.06) | 0.011^a^ | Ⅰ, n = 819 | 141 (17.22) | 678 (82.78) | 0.016^a^ |
| Ⅱ, n = 6569 | 854 (13.00) | 5715 (87.00) |  | Ⅱ, n = 6569 | 758 (11.54) | 5811 (88.46) |  | Ⅱ, n = 6569 | 883 (13.44) | 5686 (86.56) |  |
| Ⅲ, n = 9597 | 1098 (11.44) | 8499 (88.56) |  | Ⅲ, n = 9597 | 991 (10.33) | 8606 (89.67) |  | Ⅲ, n = 9597 | 1247 (12.99) | 8350 (87.01) |  |
| Ⅳ, n = 2387 | 271 (11.35) | 2116 (88.65) |  | Ⅳ, n = 2387 | 283 (11.86) | 2107 (88.27) |  | Ⅳ, n = 2387 | 306 (12.82) | 2081 (87.18) |  |
| Age, y |  |  |  | Age, y |  |  |  | Age, y |  |  |  |
| 10-17, n = 3306 | 331 (10.01) | 2975 (89.99) | <0.001^b^ | 10-17, n = 3306 | 271 (8.20) | 3035 (91.80) | <0.001^b^ | 10-17, n = 3306 | 331 (10.01) | 2975 (89.99) | <0.001^b^ |
| 18-34, n = 4582 | 564 (12.31) | 4018 (87.69) |  | 18-34, n = 4582 | 540 (11.79) | 4042 (88.21) |  | 18-34, n = 4582 | 639 (13.95) | 3943 (86.05) |  |
| 35-49, n = 4307 | 622 (14.44) | 3685 (85.56) |  | 35-49, n = 4307 | 537 (12.47) | 3770 (87.53) |  | 35-49, n = 4307 | 667 (15.49) | 3640 (84.51) |  |
| 50-64, n = 3617 | 421 (11.64) | 3196 (88.36) |  | 50-64, n = 3617 | 412 (11.39) | 3205 (88.61) |  | 50-64, n = 3617 | 437 (12.08) | 3180 (87.92) |  |
| ≥65, n = 3560 | 403 (11.32) | 3157 (88.68) |  | ≥65, n = 3560 | 378 (10.62) | 3182 (89.38) |  | ≥65, n = 3560 | 503 (14.13) | 3057 (85.87) |  |
| Gender |  |  |  | Gender |  |  |  | Gender |  |  |  |
| M, n = 9307 | 1152 (12.38) | 8155 (87.62) | 0.228 | M, n = 9307 | 1081 (11.61) | 8226 (88.39) | 0.014^a^ | M, n = 9307 | 1192 (12.81) | 8115 (87.19) | 0.051 |
| F, n = 10065 | 1189 (11.81) | 8876 (88.19) |  | F, n = 10065 | 1057 (10.50) | 9008 (89.50) |  | F, n = 10065 | 1385 (13.76) | 8680 (86.24) |  |
| Occupation |  |  |  | Occupation |  |  |  | Occupation |  |  |  |
| Stu., n = 4109 | 429 (10.44) | 3680 (89.56) | <0.001^b^ | Stu., n = 4109 | 422 (10.27) | 3687 (89.73) | 0.191 | Stu., n = 4109 | 435 (10.59) | 3674 (89.41) | <0.001^b^ |
| Tch., n = 1132 | 139 (12.28) | 993 (87.72) |  | Tch., n = 1132 | 123 (10.87) | 1009 (89.13) |  | Tch., n = 1132 | 123 (10.87) | 1009 (89.13) |  |
| Med., n = 1272 | 386 (30.35) | 886 (69.65) |  | Med., n = 1272 | 158 (12.42) | 1114 (87.58) |  | Med., n = 1272 | 274 (21.54) | 998 (78.46) |  |
| Gov., n = 591 | 101 (17.09) | 490 (82.91) |  | Gov., n = 591 | 83 (14.04) | 508 (85.96) |  | Gov., n = 591 | 102 (17.26) | 489 (82.74) |  |
| Ent., n = 876 | 125 (14.27) | 751 (85.73) |  | Ent., n = 876 | 103 (11.76) | 773 (88.24) |  | Ent., n = 876 | 122 (12.93) | 754 (86.07) |  |
| Staff, n = 6613 | 658 (9.95) | 5955 (90.05) |  | Staff, n = 6613 | 727 (10.99) | 5886 (89.01) |  | Staff, n = 6613 | 893 (13.50) | 5720 (86.50) |  |
| Framer, n = 1081 | 113 (10.45) | 968 (89.55) |  | Framer, n = 1081 | 121 (11.19) | 960 (88.81) |  | Framer, n = 1081 | 121 (11.19) | 960 (88.81) |  |
| Other, n = 795 | 96 (12.08) | 699 (87.92) |  | Other, n = 795 | 91 (11.45) | 704 (88.55) |  | Other, n = 795 | 102 (12.83) | 693 (87.17) |  |
| None, n = 2903 | 294 (10.13) | 2609 (89.87) |  | None, n = 2903 | 310 (10.68) | 2593 (89.32) |  | None, n = 2903 | 405 (13.95) | 2498 (86.05) |  |
| Education |  |  |  | Education |  |  |  | Education |  |  |  |
| Primary, n = 876 | 84 (9.59) | 792 (90.41) | 0.081 | Primary, n = 876 | 89 (10.16) | 787 (89.84) | 0.304 | Primary, n = 876 | 112 (12.79) | 764 (87.21) | 0.319 |
| Junior, n = 4589 | 582 (12.68) | 4007 (87.32) |  | Junior, n = 4589 | 504 (10.98) | 4085 (89.02) |  | Junior, n = 4589 | 601 (13.10) | 3988 (86.90) |  |
| Senior, n = 8528 | 1031 (12.09) | 7497 (87.91) |  | Senior, n = 8528 | 917 (10.75) | 7611 (89.25) |  | Senior, n = 8528 | 1142 (13.39) | 7386 (86.61) |  |
| Uni., n = 5379 | 644 (11.97) | 4735 (88.03) |  | Uni., n = 5379 | 628 (11.68) | 4751 (88.32) |  | Uni., n = 5379 | 722 (13.42) | 4657 (86.58) |  |
| Marriage |  |  |  | Marriage |  |  |  | Marriage |  |  |  |
| Unm., n = 5741 | 648 (11.29) | 5093 (88.71) | 0.077 | Unm., n = 5741 | 642 (11.18) | 5099 (88.82) | 0.007^b^ | Unm., n = 5741 | 762 (13.27) | 4979 (86.73) | 0.237 |
| M., n = 13002 | 1611 (12.39) | 11391 (87.61) |  | M., n = 13002 | 1401 (10.78) | 11601 (89.22) |  | M., n = 13002 | 1719 (13.22) | 11283 (86.78) |  |
| Div., n = 629 | 82 (13.04) | 547 (86.96) |  | Div., n = 629 | 95 (15.10) | 534 (84.90) |  | Div., n = 629 | 96 (15.26) | 533 (84.74) |  |

GAD-7: the Generalized Anxiety Disorder-7 scale; PHQ-9: the Patient Health Questionnaire-9; ISI: the Insomnia Severity Index; M: Male; F: Female; Stu.: Student; Tch.: Teacher; Medical; Gov.: Government; Ent.: Enterprise; Uni.: University and above; Unm.: Unmarried; M.: Married; Div.: Divorced. * For participants aged under 18, scores ≥11 indicate depression; ^a^*p* < 0.05 (Univariate logistic regression); ^b^*p* < 0.01 (Univariate logistic regression).
